# Implementation of Intelligent Physical Exercise Training at a Danish Hospital—A Qualitative Study of Employees’ Barriers and Facilitators for Participation

**DOI:** 10.3390/ijerph20227085

**Published:** 2023-11-20

**Authors:** Christina Juul Pultz, Thea Mundt Lohse, Just Bendix Justesen, Charlotte Ahlgren Særvoll, Sofie Fønsskov Møller, Birgitte Lindegaard, Thea K. Fischer, Tina Dalager, Stig Molsted

**Affiliations:** 1 Department of Sports Science and Clinical Biomechanics, The Faculty of Health Science, University of Southern Denmark, 5000 Odense, Denmark; cjpultz@gmail.com (C.J.P.); thea.mulo@gmail.com (T.M.L.); tdalager@health.sdu.dk (T.D.); 2 Department of Sports Science and Clinical Biomechanics, Research Unit of Physical Activity and Health in Working Life, University of Southern Denmark, 5000 Odense, Denmark; jbjustesen@health.sdu.dk; 3 Department of Clinical Research, Copenhagen University Hospital—North Zealand, 3400 Hillerød, Denmark; charlotte.ahlgren.saervoll@regionh.dk (C.A.S.); sofiefoensskovmoeller@gmail.com (S.F.M.);; 4 Department of Pulmonary and Infectious Diseases, Copenhagen University Hospital—North Zealand, 3400 Hillerød, Denmark; birgitte.lindegaard.madsen@regionh.dk; 5 Centre for Physical Activity Research, Copenhagen University Hospital—Rigshospitalet, 2100 Copenhagen, Denmark; 6 Department of Public Health, University of Copenhagen, 1172 København, Denmark; 7 Department of Clinical Medicine, Faculty of Health, Aarhus University, 8000 Aarhus, Denmark; 8 Department of Clinical Medicine, Faculty of Health and Medical Sciences, University of Copenhagen, 1172 København, Denmark

**Keywords:** employees, interview, physical activity, qualitative research, workplace

## Abstract

Background: Exercise training at work has the potential to improve employees’ productivity, health, and well-being. However, exercise interventions for healthcare workers in hospitals may be challenged by time pressure and the ongoing workflow with patient care. Objective: The aim was to identify barriers and facilitators for participation in exercise training during work in a hospital department. Methods: Eight semi-structured interviews of 13 individuals were conducted with hospital employees from different staff groups who participated in 12 weeks of exercise twice weekly. The data analysis was a thematic approach based on the Theoretical Domains Framework and the COM-B factors in the Behavior Change Wheel. Results: Barriers and facilitators varied between different groups. Barriers included limited structure, busyness, and a discouraging culture. Facilitators included gaining a feeling of community and psychological and physical well-being. Seven contextual subthemes were vital for successful implementation of exercise in a hospital setting: sharing of knowledge and information; involvement; administration and structure; culture; individualization; purpose and objective; and incentives. Conclusions: The informants appreciated exercise training during work. Inpatient departments’ informants found it difficult to participate in the intervention, whilst those with more administrative tasks found it easier. This study identified barriers and facilitators vital for a successful implementation of an exercise training intervention in a hospital department. The study explains how future interventions can improve reach, adoption, and implementation of exercise training interventions to hospital staffs.

## 1. Introduction

More than one third of the adult population in western, high-income countries is physically inactive [1,2,3]. Physical inactivity leads to increased risk of chronic diseases, obesity, and early death [4]. Conversely, physical activity has been proven to prevent chronic diseases and to increase mental health, quality of life, and well-being [4,5,6].

During the last decades, more workplaces provide physical exercise training during work, and the workplace is considered an ideal arena where physical exercise training programs can be implemented [7,8]. However, despite promising effects of exercise, one size usually does not fit all, and a personalized exercise program my lead to greater effects. Sjøgaard and colleagues [9] recently developed and published the concept of ‘Intelligent Physical Exercise Training’ (IPET), which is individualized physical exercise training that consists of 60 min, weekly, moderate-to-high intensity training tailored to each employee’s work exposure and individual health profile. Dalager and colleagues [10] demonstrated the effectiveness of IPET on aerobic capacity and blood pressure in office workers. Furthermore, IPET has also increased workability and productivity and decreased sickness-related absenteeism by 29% in office workers [11].

Previous studies report that participation in exercise training at workplaces can be challenging. A review of nine Danish randomized controlled trials found that continuous adherence to physical activity at the workplace varied from 31–86% [12]. Jørgensen and colleagues [13] examined factors associated with low participation in health-promoting activities at the workplace and found that the high physical and/or psychological demands of the job, combined with low job control, reduced participation. In an intervention study, Ilvig and Bredahl [14] tried to implement IPET at a workplace in a healthcare context and also faced challenges: female healthcare workers employed in home care and nursing homes reported that the reduced flexibility at the workplace, the lack of support from management, the content and intensity of the programs, and the low coherence between published information and the reality of the workplace were barriers to participation. Everyday life in a hospital can be unpredictable and changeable, as demands are constantly placed on the staff from patients and relatives to provide care [15]. Moreover, staffs in Danish hospitals are under great pressure as a result of understaffing, and among nurses, up to 60% have reported that they felt stressed and blamed work as the underlying reason [16], which could be a barrier to participate in exercise during work. In addition, nurses have reported a high prevalence of musculoskeletal pain, which entails increased risk of long-term sick leave [17]. Thus, there is a high need to intervene within this profession and with other healthcare workers. Exercise during work may be an important intervention to enhance the resilience of healthcare workers and improve their health.

This qualitative study was conducted during a pilot trial of IPET during working hours in a hospital department [18]. The intervention showed positive changes of objectively and subjectively measured health outcomes, and data on clinical health parameters, well-being, productivity, and self-rated health from the pilot trail have been published elsewhere [18]. However, the adherence was low, and the dropout rate was highest among nurses and social and healthcare assistants [18]. Even though IPET during work is associated with positive changes in a hospital, more knowledge is needed to identify barriers and facilitators to prevent dropout and low adherence. Thus, the aim of this study was to identify barriers and facilitators for participation in an IPET intervention during working hours.

## 2. Methods

### 2.1. Design

The study was a qualitative study conducted with a sample of employees who participated in a pilot trial at Department of Pulmonary and Infectious Disease, Nordsjælland’s Hospital, Denmark, from August to December 2021. Semi-structured interviews were conducted to elucidate the employees’ barriers and facilitators.

### 2.2. Ethics

The informants gave oral and written consent before the interviews. The study complied with The Central Capital Region Committees on Health Research Ethics (H-21038302) and The Data Protection Agency (P-2021-472), and the study was performed in accordance with the Helsinki Declaration.

### 2.3. The Intervention

A description of the intervention is published elsewhere [18]. In brief, the IPET intervention was free of charge and lasted for 12 weeks. The employees were invited to participate in two weekly training sessions of 30 min each during work hours from 7.30 AM to 3.45 PM in which several exercise sessions were provided. The training sessions were performed in groups of maximum 12 participants and consisted of a short warm up followed by individualized exercises within the training categories of aerobic training, resistance training, and balance training. The exercises were individualized to each employee based on a baseline health examination (aerobic capacity, blood pressure, musculoskeletal pain) and the employee’s exercise preferences. All exercise sessions were supervised by a professional educated in sport science or physiotherapy. 

### 2.4. Sampling and Recruitment

We used a structured recruitment strategy based on the level of participation in the IPET intervention, focusing on employees who (1) did not wish to participate, (2) signed up but did not participate, (3) signed up but dropped out during the first weeks, and (4) participated throughout the intervention. As participation in the intervention varied depending on profession, we sought to recruit the nursing staff, the administrative staff, and management. Recruitment for the interviews was performed by author J.B.J. and an established contact person at the hospital who provided a list of employees with information about profession and degree of participation in the intervention. 

### 2.5. Interview Process 

The eight semi-structured interviews, except one, totaled 13 individuals and were conducted face-to-face with the management, administrative, and nursing staffs from December 2021 to April 2022. The informants were given fictitious names to ensure anonymity. They were interviewed at their workplace in an undisturbed setting and with interview durations of 16–43 min.

A semi-structured interview guide was designed based on the existing literature (see Appendix A). The interview guide consisted of open-ended questions prepared with supplemental questions for elaboration. Questions were formulated using everyday language and conducted in Danish, audio-recorded, and transcribed verbatim within 24 h after the interview by author T.M.L. or C.J.P. Informants were ensured complete anonymity.

### 2.6. Data Analysis 

The structure of the analysis was based on a thematic approach containing six iterative phases: (1) familiarization, (2) coding, (3) theme development, (4) refinement, (5) naming, and (6) writing up [19]. First, familiarization with the data was achieved by thoroughly reading it and noting the most dominant content. Second, the Theoretical Domains Framework (TDF) [20] was used for deductive coding of the data and to identify patterns. The TDF is a framework of a psychological model with 14 domains. The model was developed to contribute to more successful implementations of evidence-based practices. Each domain represents different psychological and social factors that can impact human behavior. The TDF provides a structured way to code data and assess which factors are affecting a particular behavior. Third, themes were categorized according to these three factors: capability (physical and psychological), opportunity (social and physical), and motivation (automatic and reflective), which are from the COM-B model in The Behavioral Change Wheel (BCW) [21]. The COM-B model is part of the theoretical framework of BCW that focuses on behavioral changes when implementing interventions, and the BCW is a behavioral change model that assesses function of capability, opportunity, and motivation. By categorizing data into these components, the framework provides a systematic and evidence-based approach for addressing the influence of essential factors on human behavior. Fourth, the subthemes were identified in relation to specific barriers and facilitators in the context of the hospital. The subthemes were used as a basis for discussing the findings. Fifth, the domains were listed depending on which were most frequent, less frequent, and rarely mentioned. Sixth phase consisted of reading and processing the material and writing. Authors CJP and TML drafted first version of the analysis, and JBJ and TD were involved until consensus was reached. The TDF and COM-B were applied to systematize and structure the findings and create recommendations for a more successful future implementation of evidence-based practice [22,23,24]. 

## 3. Results

**Figure 1:**
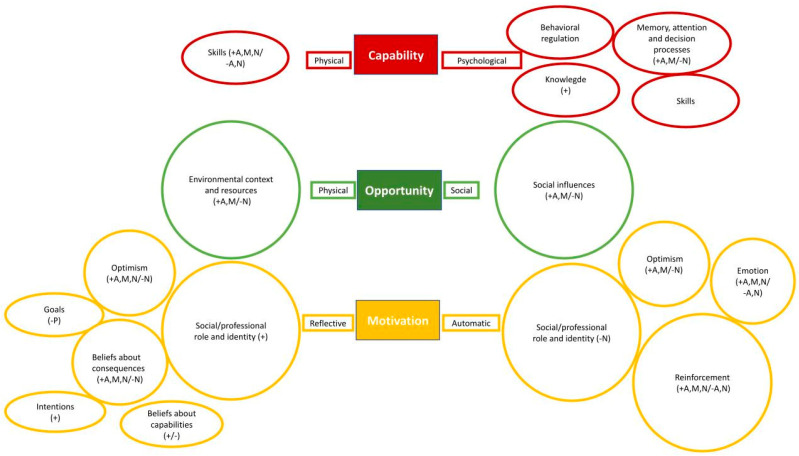
Represents an overview of the results of the thematic analysis. The TDF-domains are connected to COM-B model’s factors inspired by Cane, O´Connor [20]. Large circles are the most frequently represented codes, small circles are the moderately represented codes, and the long circles are the least frequent codes. In the parentheses, ‘+’ marks the domain as a facilitator, while ‘−’ marks it as a barrier. ‘A’ stands for ‘administrative staff’, ‘M’ stands for ‘managers’, and ‘N’ stands for ‘nursing staff’. The TDF has 14 domains; however, the Figure have 17 circles since the domains ‘skills’, ‘optimism’, and ‘social/professional role and identity’ are reported twice.

Table 1 presents the characteristics of informants and interview methods. For level of participation, seven individuals participated in 13 (2–24) (median (range)) out of 24 exercise sessions during the intervention period. Figure 1 presents an overview of the results of the thematic analysis: it represents each TDF domains’ impact by its size, which is based on the number of codes given during the analysis within the specific domains. Furthermore, the domains are connected to the COM-B factors. Whilst the TDF has 14 domains, Figure 1 presents 17 domains since three of them are reported twice.

In general, all informants found the IPET intervention during working hours relevant, and all wanted to participate in future IPET interventions. During the data collection, we found different opinions within the staff groups of what was considered crucial for participating in the intervention. We have identified subthemes linked to each COM-B factor that are crucial for implementing IPET at this specific hospital department. Table 2 provides an overview of the link between TDF domains, COM-B factors, and the subthemes identified by the authors. 

### 3.1. Capability

Based on the analysis of ‘capability’, two subthemes related to the context of the hospital department were identified: ‘sharing of knowledge and information’ and ‘involvement’.

#### 3.1.1. Psychological Capability

The most frequently identified barrier in ‘psychological capability’ was the coordination and planning of the training during workdays. The nursing staff experienced difficulties in being able to participate. Initially, they felt motivated to participate but found it very difficult to join the training sessions as a result of the workload.

**Nina:** *“(…) I was in favor of it in the beginning too. And I was very much like “come on friends, we’ll do it” and “we must all join” and organized one or the other department competition to see who could lose most weight and so on. Umm… But I don’t think that I will get them to participate in that again. Haha…”*

(Interview 7, l 122-125, TDF: Behavioral regulation)

Some employees felt that they did not receive information about the intervention, which became a barrier for participation. In addition, they felt that they were a piece of a larger puzzle and that the purpose of the project was related more to the increased productivity and research results than to the well-being of the employees.

**Allison:** *“Yes, I felt a bit like I was used as a test animal. I was a part of an initiative to obtain research results.”*

(Interview 4, l 598-599, TDF: Knowledge)

Facilitators regarding ’psychological capability’ included having a personalized exercise program and being able to coordinate who was exercising in the entire unit. Furthermore, the information provided regarding the intervention from the study workers facilitated participation. 

#### 3.1.2. Physical Capability

Some participants experienced limitations in their ‘physical capability’ because the intervention was too vigorous and provoked previous injuries.

**Allison:** *“It was simply too hard. Because… one would say that with the type of injury I have (…), it is really important for me to have a lot of stability exercises… (…) Even though there were, the exercises were too hard.”*

(Interview 4, l 29-31, TDF: Skills)

Other participants felt that their ‘physical capability’ was improved by the challenging and tailored exercise programs that took individual needs into consideration.

**Amber:** *“I can also say that…, for example… I’ve got diagnosed (disease). So, I have simply been pleased by the fact that some people have gone to great lengths to find things for me, and I had special programs tailored for me.”*

(Interview 3, l 111-114, TDF: Skills)

Some informants described that they felt involved in choosing what kinds of exercises they should do, while others felt like the exercise program was not individualized enough. 

### 3.2. Opportunity

Through the analysis of physical and social opportunity, two subthemes related to the context of the hospital department were identified: ‘administration and structure’ and ‘culture’.

#### 3.2.1. Physical Opportunity

The most frequent barrier in ‘physical opportunity’ was the pressure from busyness in the department. Patients in need of care would always be a priority. While the managers and administrative staff were able to coordinate duty schedules and participate in the intervention, the nursing staff often found it difficult to leave the department to take part in the IPET.

**Nina:** *“But it just ended up with you suddenly being responsible for eight patients instead of four or something, right? Because one of your colleagues had to leave. (…) when you then were given the responsibility for someone else’s patients for an hour, during the visitation of these patients, it was difficult to have to follow up on eight patients (…) Those who are not part of the actual staffing and those who are not working in the care departments, they can find time for it and sort of structure it and plan it accordingly.”*

(Interview 7, l 23-33, TDF: Environmental context and resources)

**Natalie:** *“I also think those who were training (…) were the quality nurse and the intro nurse. It was those people, who don’t have patients or not that many patients… or those with an intern, if you can put it that way.”*

(Interview 6, l 36-38, TDF: Environmental context and resources)

The pressure of busyness was also evident regarding when the interviews should be scheduled. We experienced difficulties in setting up meetings with the nursing staff. However, one of the managers suggested that the busyness in the department had more to do with the employees being exhausted after the COVID-19 pandemic than the actual busyness.

**Megan:** *“But what we can see in this hospital is, that it is not busy, we do not have high occupancy, all the beds are not occupied every day. And we have not cut down in staffing, there has not been cutbacks in several years. And the vacant positions they have, has not increased a lot the last couple of years. On the contrary, almost no one has gotten all their staff hired (…) and because it has been busy, and COVID came along, and they are maybe tired. Then that exhaustion, is what we need to talk to them about. And that has something to do with staff management.”*

(Interview 8, l 164-170, TDF: Environmental context and resources)

With regard to ‘physical opportunity’, the experiences of the managers and administrative staff revealed that structuring and administration of the workday were facilitators for exercising. 

#### 3.2.2. Social Opportunity 

Regarding ‘social opportunity’, social pressure was reported as a barrier. The administrative and nursing staffs felt that they imposed a greater workload on their coworkers when they left the department to exercise, which resulted in irritation and a negative atmosphere.

**Amber:** *“Yes, because I’ve actually heard something… someone saying “Well, I can’t go train because I have to look after yours. I can’t go”. It will very quickly create friction.”*

(Interview 3, l 181-183, TDF: Social influence)

Facilitators for ‘social opportunity’ were a sense of coherence, doing something differently than working with colleagues, and getting to know coworkers from other departments.

**Amber:** *“The fact that we are a large department, spread over many departments, that you actually also met each other in another setting. So, it benefited both yourself and the group. Someone you might not have seen in months, right? The thing about training together.”*

(Interview 3, l 49-52, TDF: Social influence)

The statements from the staff suggest that the staff might benefit from participating in IPET but also that there might be a culture within the department that makes it difficult to leave to participate. 

### 3.3. Motivation

Based on the results of the analysis of ‘motivation’, three subthemes were identified: ‘individualization’, ‘purpose and goalsetting’, and ‘incentives’.

#### 3.3.1. Reflective Motivation

Barriers regarding ‘reflective motivation’ covered not having a promised physical test conducted before the intervention and little regard or consideration to previous injuries. Furthermore, the nursing staff did not believe that implementing the intervention during working hours was realistic. The nursing staff viewed the intervention as a promotion of the department by top management and as a way of doing something good for them as staff, but in a misunderstood way. They felt that the purpose was to optimize the workflow at the department by trying to make the staff more productive instead of taking a critical look at workplace conditions and having enough staff at work.

**Natalie:** *“I also think that it is difficult that we now also have to do that. We are constantly pulled into this or that or the other project. And the managers keep saying: “It’s a good idea, and we would like to be contributing to that” and good God. But it’s just not always that the circumstances or the resources then follow (…). But the time is also not provided, even if it is supported by the section management and department management.”*

(Interview 6, l 255-260, TDF: Social/professional role and identity)

**Nicole:** *“Then you have to go out yourself and make some kind of extra effort, you actually don’t want to. So, it just becomes very manipulative in one way or another and it appears as though we have to in order for us to attract new employees and… because we train during working hours. (…) The speed at which articles came out, to tell how crazy good we are here, because the management allows us to train. And then it’s all just chaos, and you can’t get anyone to take care of your patients and stuff like that.”*

(Interview 6, l 544-549, TDF: Optimism)

The nursing staff found that the goals of the intervention were too vague; thus, they found it difficult to set their own goals. 

Facilitators of the ‘reflective motivation’ were tailored and individualized exercise training programs, and the training prescription was 2 × 30 min a week, which was a foreseeable period. The managers tried to enhance their staff’s ‘reflective motivation’ by participating in the intervention themselves. 

#### 3.3.2. Automatic Motivation

With regard to ‘automatic motivation’ (automatic processes, including impulses and inhibition), there were a lack of role models. The project team had planned to educate ‘training ambassadors’ to facilitate the implementation. Some informants pointed out that involving training ambassadors in the implementation process could have increased the staff’s motivation for participating in the training.

**Madison:** *“So maybe they should have been here more. Those who trained (the exercise experts). That is, in the morning and try to get people along or… walk around during the day and talk to people once in a while and drop by a little bit. They dropped by a few times, but it was very little. But stay here a little longer and try to pull people along a bit too, so that they… “give it a go” or “is there something that prevents you?”, “can we try that?”.”*

(Interview 5, l 134-138, TDF: Reinforcement)

The staff groups working in inpatient sections of the departments found it easier to attend the training sessions held in the morning and afternoon rather than the sessions held midday. Some of the nursing staff found the training to be dull and monotonous.

The employees believed that the managers tried to make it possible for them to leave the department to exercise but did not provide the right work conditions to do so.

**Nicole:** *“But I also know, from the management. They also frequently tried to state that we should try to let people go down and try because it is important that you participated. But then there was such an obvious irritation… and then again, we also think it is annoying when you get more tasks.”*

(Interview 6, l 73-76, TDF: Emotions)

The intervention’s positive effects on mood, health, and work productivity served as facilitators in ‘automatic motivation’. Furthermore, the exercise experts and the setting of the intervention were considered motivational for the management and administrative staffs.

**Marc:** *“But among those who are still in, there is some kind of dynamic and joy. There is… you do things, the task in a different way. So, in reality I think, that this is one of the really big benefits.”*

(Interview 3, l 507-510, TDF: Optimism)

**Amber:** *“Well, our manager led by example. And you also trained together with them occasionally, and it was really nice to meet your manager in a different way.”*

(Interview 3, l 546-548, TDF: Social/professional role and identity, Emotions)

Table 2 provides an overview of identified subthemes in relation to TDF domains and COM-B factors.

## 4. Discussion

This study identified barriers and facilitators to the participation in exercise training during work at a hospital department. Barriers included the limited structure of the workday, which made it difficult to leave the department to exercise, and the insufficient facilitation of participation in the training. Facilitators of IPET included feelings of physical and psychological well-being, motivating exercise programs and exercise experts, a sense of community through exercising, and management’s assistance with coordinating and structuring the workday to make time for participation in IPET.

### 4.1. Structure and Involvement

The nursing staff, managers, and administrative staff showed a desire to participate in the intervention. However, nurses especially felt incapable of participating due to time pressure and the ongoing workflow. The nurses felt that their work schedule, including unpredictable tasks, was a barrier to participation, and they feared that patient safety could be compromised. Indeed, unpredictable tasks, in combination with time pressure, may reduce nurses’ job autonomy and thus reduce their motivation to participate in the intervention. Kirk, Sivertsen [25] found the same challenges during the implementation of a screening tool in a Danish acute care unit. Limited resources, including time, led to the staff being afraid of making mistakes, thereby influencing patient safety negatively. Kirk, Sivertsen [25] emphasized the managers’ roles as facilitators in cases where they chose to support the desired change. In this study, the nurses wanted more involvement and cooperation from management to create structure in their work to make it possible to leave the department to exercise. A systematic review found that management could function as a barrier as well as a facilitator during the change process, depending on whether they were supportive or absent [26]. For successful implementation, management is supposed to endorse the intervention, understand its relevance, and provide the necessary flexibility during workdays. To support the nursing staff’s participation in the intervention, management might consider assisting them with structure in their workday and to be clearly supportive of the desired change.

The managers and administrative staff expressed how clear communication, including the presentation of test results, could be a determinant for successful implementation. Chigumete, Townsend [27] found that the poor communication and inadequate involvement of employees were barriers to the implementation of health-promoting initiatives in a South African hospital. The nursing and administrative staffs pointed out that it might have been relevant to further involve the nurses in the development and planning of the intervention. It might also be important for management to take greater responsibility for communicating the hospital’s vision and purpose of the implementation, which may lead to greater employee involvement in the design and planning of the intervention, instead of expecting the communication to be delivered by others. Involving the employees might create a sense of ownership regarding the intervention and elevate the staff’s motivation to participate in the intervention. 

### 4.2. Work Culture

There was a negative attitude among some nurses towards the intervention. This attitude included irritation and limited understandings when a colleague participated in the intervention. Studies have shown that a culture without support for a new approach can be a barrier to participation in health-promoting activities [12,23,24,26]. The successful implementation of a health-promoting intervention may require legitimacy from management, participators, and colleagues. A positive atmosphere and a joint effort among colleagues may reduce sedentary behavior at work [28].

The study highlighted different perceptions of how busy the department was. While some employees indicated that they did not have time to participate in IPET, management indicated that there was not full occupancy in the department. Recently, the implementation of a major IT platform, the COVID-19 pandemic, and a nursing strike in the late summer of 2021 in Denmark negatively affected the cooperation and well-being of the Danish healthcare staff [29,30]. Figures from 2018 show a similar trend, and the workplace pressure in Danish hospitals was an issue before COVID-19 [31]. This leads to uncertainty as to whether there are work assignments that are invisible to management, including difficult conversations with patients and time-consuming hygiene tasks. Both examples are quality healthcare tasks that are difficult to measure compared to quantitative measures, such as occupancy. Furthermore, the busyness and pressure could create a culture in which employees are not able to participate in training during the workday.

### 4.3. Health Ambassadors as Change Agents

When the study was planned, the research and project groups considered utilizing health ambassadors to facilitate participation in the intervention; however, this was not carried out. Change agents can be equated with the aforementioned health ambassadors. Utilizing change agents in the study might have aided management and the study groups. The management and project groups could control the project, while the change agents could focus on facilitating the desired behavioral changes. Daniels, Watson [26] emphasized the importance of securing continuity in the change process, which health ambassadors could possibly assist with.

Choosing health ambassadors among the staff and making sure they are accepted by the rest of the group may save resources and gain credibility more easily. If health ambassadors are chosen as part of a future intervention, it would be important to structure their workday, give them time, and make it possible for them to succeed in their new positions. Ensuring that the health ambassadors have the right skills and offering them education in change processes could be essential for their role [32].

### 4.4. Implications for Occupational Health Practice

The results of this study are important to consider in the implementation of IPET during working hours in hospitals. Implementing IPET at a hospital requires consideration for the nurses’ and other employees’ workflow and motivations to participate. All participants valued the communal feelings among colleagues but missed communication about the intervention and management support regarding adjusting the workflow to accommodate the implementation of IPET. 

In future interventions, the reconciliation of expectations between management and staff is important. Furthermore, it is essential to involve the staff in the design of the intervention before IPET is implemented. Likewise, long-term, successful implementation demands a cultural change. The health ambassadors could contribute by driving this change with a positive attitude. This means that when starting the next intervention, management should look not only at the short-term effects but also the long-term perspectives.

### 4.5. Strengths and Limitations 

One strength of this study is that the interviews were conducted with multiple staff groups within a hospital department, which allowed the inclusion of different views of the implementation process. We experienced data saturation during our interviews because the informants covered the same topics from different angles. However, we acknowledge the low number of informants. The planned focus groups often turned into single interviews, which gave the interviews a different setup than what was initially planned. This meant that we could ask the informants to elaborate on their answers. 

## 5. Conclusions

Overall, the informants liked the idea of physical exercise training during working hours. Experienced barriers and facilitators varied among the included staff members. Informants from inpatient departments had more difficulties participating in the intervention, whilst those working with more administrative tasks found it easier to prioritize participation. Managerial support for and assistance to the staff when their workdays needed to be structured differently and staff involvement throughout the entire implementation process were found to be essential for success. Furthermore, it was important that employees and managers supported the intervention, which created a culture that facilitated participation.

## Figures and Tables

**Table 1:** Overview of conducted interviews. Names starting with ‘M’ indicate managing staff. Names starting with ‘A’ indicate administrative staff. Names starting with ‘N’ indicate nursing staff.

Participant’s Names	Interview Type	Interviewer	Informant’s Position	Informant’s Participation in the Project
Mary	Single (planned as focus group)	Project manager 2 master’s students	Manager	Did not participate
Michael	Single (planned as focus group)	Project manager 2 master’s students	Manager	Participated during most of the project but had to stop due to injury
Ann Amy Amber Marc	Focus group	Project manager 1 master’s student	Administrative staff (*n* = 3) Manager (*n* = 1)	Participated through the entire project
Nick Allison	Focus group/ two single interviews	Project manager 1 master’s student	Nursing staff (*n* = 1) Administrative staff (*n* = 1)	1 stopped mid-project due to injury. 1 stopped participation due to time pressure.
Madison	Single	2 master’s students	Manager	Participated through the entire project
Nicole Natalie	Two single interviews	2 master’s students	Nursing staff	1 participated through the entire project. 1 stopped mid-project.
Nina	Single by phone	2 master’s students	Nursing staff	Participated twice
Megan	Single	Project manager 1 master’s student	Manager	Did not participate

**Table 2:** Overview of identified subthemes in relation to TDF domains and COM-B factors.

TDF Domain	COM-B Factor	Subtheme
**Skills** **Behavioral regulation** **Memory, attention, and decision processes** **Knowledge**	Capability	Sharing of knowledge and information Involvement
**Environmental context and resources** **Social influences**	Opportunity	Administration and structure Culture
**Goals** **Optimism** **Intentions** **Beliefs about consequences** **Beliefs about capabilities** **Social/professional role and identity** **Emotions** **Reinforcement**	Motivation	Individualization Purpose and objective Incentives

The TDF domains are related to the different COM-B factors and subthemes.

## Data Availability

The data are available by request to the study group.

## Appendix A

**Interview guide**

Research questions

Based on change management, the implementation of a health intervention at Nordsjælland’s Hospital is examined with a view that includes how the role of middle managers influences the implementation of the intervention.

1. What is the culture in the department in relation to physical activity during working hours?
2. What type of culture is required in the department to support continued physical activity during working hours?
3. What are the experiences of employees and middle managers during the implementation process?
4. What do middle managers experience as barriers and motivation during the implementation process?

Organizational culture

How can the mood and atmosphere change depending on how these roles are played and whether new roles are introduced in relation to the intervention of physical activity during working hours?

Other things

1. Do the individuals like how the physical activity is structured?
2. Who are the various participants (the managers specifically/the change agents/senior management)?

## References

[1] Guthold R., Stevens G.A., Riley L.M., Bull F.C. (2018). Worldwide Trends in Insufficient Physical Activity from 2001 to 2016: A Pooled Analysis of 358 Population-based Surveys with 1.9 Million Participants. Lancet Glob. Health.

[2] Rasmussen L.B., Andersen L.F., Borodulin H.E.B., Fagt S., Matthiessen J., Sveinsson T., Thorgeirsdottir H., Trolle E. (2011). Nordic Monitoring of Diet, Physical Activity and Overweight: First Collection of Data in Alle Nordic Countries.

[3] Pleis J.R., Ward B.W., Lucas J.W. (2010). Summary health statistics for U.S. adults: National Health Interview Survey. Vital Health Stat..

[4] Pedersen B.K. (2019). The Physiology of Optimizing Health with Focus on Exercise as Medicine. Annu. Rev. Physiol..

[5] Sanchis-Gomar F., Lavie C.J., Marín J., Perez-Quilis C., Eijsvogels T.M.H., O’keefe J.H., Perez M.V., Blair S.N. (2022). Exercise effects on cardiovascular disease: From basic aspects to clinical evidence. Carsiovasc Res..

[6] Mikkelsen K., Stojanovska L., Polenakovic M., Bosevski M., Apostolopoulos V. (2017). Exercise and mental health. Maturitas.

[7] Kuoppala J., Lamminpää A., Husman P. (2008). Work health promotion, job well-being, and sickness absences—A systematic review and meta-analysis. J. Occup. Environ. Med..

[8] Robroek S.J., van Lenthe F.J., van Empelen P., Burdof A. (2009). Determinants of participation in worksite health promotion programmes: A systematic review. Int. J. Behav. Nutr. Phys. Act..

[9] Sjøgaard G., Justesen J.B., Murray M., Dalager T., Søgaard K. (2014). A Conceptual Model for Worksite Intelligent Physical Exercise Training—IPET—Intervention for Decreasing Life Style Health Risk Indicators among Employees: A Randomized Controlled Trial. BMC Public Health.

[10] Dalager T., Justesen J.B., Murray M., Boyle E., Sjøgaard G. (2016). Implementing Intelligent Physical Exercise Training at the Workplace: Health Effects Among Office Workers—A Randomized Controlled Trial. Eur. J. Appl. Physiol..

[11] Justesen J.B., Søgaard K., Dalager T., Christensen J.R., Sjøgaard G. (2017). The Effect of Intelligent Physical Exercise Training on Sickness Presenteeism and Absenteeism among Office Workers: A Randomized Controlled Trial. J. Occup. Environ. Med..

[12] Garne-Dalgaard A., Mann S., Bredahl T.V.G., Stochkendahl M.J. (2019). Implementation Strategies, and Barriers and Facilitators for Implementation of Physical Activity at Work: A Scoping Review. Chiropr. Man. Ther..

[13] Jørgensen M.B., Villadsen E., Burr H., Punnett L., Holtermann A. (2016). Does Employee Participation in Workplace Health Promotion Depend on the Working Environment? A Cross-sectional Study of Danish Workers. BMJ Open.

[14] Ilvig P.M., Bredahl T.V.G., Justensen J.B., Jones D., Lundgaard J.B., Søgaard K., Christensen J.R. (2018). Attendance Barriers Experienced by Female Health Care Workers Voluntarilty Participating in a Multi-component Health Promotion Programme at the Workplace. BMC Public Health.

[15] Nielsen H.B., Hansen M., Conway S.H., Dyreborg J., Hansen J., Kolstad H.A., Larsen A.D., Nabe-Nielsen K., Pompeii L.A., Garde A.H. (2019). Short Time Between Shifts and Risk of Injury among Danish Hospital Workers: A Register Based Cohorte Study. Scand. J. Work Environ. Health.

[16] Dansk Sygeplejeråd (2021). SATH Undersøgelse—Sygeplejerskers Arbejdsmiljø, Trivsel og Helbred. https://dsr.dk/loen-og-arbejdsvilkaar/arbejdsmiljoe/undersoegelser-af-arbejdsmiljoe-sath/.

[17] Andersen L.L., Clausen T., Carneiro I.G., Holtermann A. (2012). Spreading of Chronic Pain between Body Regions: Prospective Cohorte Study among Health Care Workers. Eur. J. Pain.

[18] Molsted S., Justesen J.B., Møller S.F., Særvoll C.A., Krogh-Madsen R., Pedersen B.K., Fischer T.K., Dalager T., Lindegaard B. (2022). Exercise Training during Working Hours at a Hospital Department: A Pilot Study. J. Occup. Eviron. Med..

[19] Braun V., Clarke V., Weate P., Smith B., Sparkes A.C. (2019). Using Thematic Analysis in Sport and Exercise Research. Routledge Handbook of Qualitative Research in Sport and Exercise.

[20] Cane J., O’Connor D., Michie S. (2012). Validation of the Theoretical Domains Framework for Use in Behaviour Change and Implementation Research. Implement. Sci..

[21] Michie S., van Stralen M.M., West R. (2011). The Behaviour Change Wheel: A New Method for Characterising and Designing Behaviour Change Interventions. Implement. Sci..

[22] Francis J.J., Stockton C., Eccles M.P., Johnston M., Cuthbertson B.H., Grimshaw J.M., Hyde C., Tinmouth A., Stanworth S.J. (2009). Evidense-based Selection of Theories for Designing Behaviour Change Interventions: Using Methods Based on Theoritical Construct Domains to Understand Clinician’s Blood Transfusion Behaviour. Br. J. Health Psychol..

[23] Munir F., Biddle S.J.H., Davies M.J., Dunstan D., Esliger D., Gray L.J., Jackson B.R., O’connell S.E., Yates T., Edwardson C.L. (2018). Stand More at Work (SMArT Work): Using the Behaviour Change Wheel to Develop an Intervention to Reduce Sitting Time in the Workplace. BMC Public Health.

[24] Ojo S.O., Bailey D.P., Brierley M.L., Hewson D.J., Chater A.M. (2019). Breaking Barriers: Using the Behavior Change Wheel to Develop a Tailored Intervention to Overcome Work Place Inhibiters to Breaking Up Sitting Time. BMC Public Health.

[25] Kirk J.W., Sivertsen D.M., Petersen J., Nilsen P., Petersen H.V. (2016). Barriers and Facilitators for Implementing a New Screening Tool in an Emergency Department: A Qualitative Study Applying the Theoretical Domains Framework. J. Clin. Nurs..

[26] Daniels K., Watson D., Nayani R., Tregaskis O., Hogg M., Etuknwa A., Semkina A. (2021). Implementing Practices Focused on Workplace Health and Psychological Wellbeing: A Systematic Review. Soc. Sci. Med..

[27] Chigumete T.G., Townsend N., Srinivas S.C. (2018). Facilitating and Limiting Factors of Workplace Promotion at Rhodes University, South Africa. Work.

[28] Danquah I.H., Kloster S., Tolstrup J.S. (2020). “Oh-oh, the Others are Standing Up… I Better Do the Same”. Mixed-method Evaluation of the Implementation Process of ‘Take a Stand!’—A Cluster Randomized Controlled Trial of a Multicomponent Intervention to Reduce Sitting Time Among Office Workers. BMC Public Health.

[29] Dansk Sygeplejeråd (2021). Dansk Sygeplejeråds Årsrapport 2021. https://dsr.dk/om-dsr/arsrapporter-fra-dansk-sygeplejeraad/.

[30] Hagedorn-Rasmussen P., Clausen T., Abildgaard J.S., Aust B., Grønvad M.T., Lund H.L., Thomsen R. (2021). Psykosocialt Arbejdsmiljø På Regionale Arbejdspladser—En Kortlægningsrapport. https://nfa.dk/da/Forskning/Udgivelse?journalId=8e60ea12-597b-40e9-80ae-3cf2a7bf0601.

[31] Dansk Sygeplejeråd (2018). SATH Undersøgelse—Sygeplejerskers Arbejdsmiljø, Trivsel og Helbred. https://dsr.dk/loen-og-arbejdsvilkaar/arbejdsmiljoe/undersoegelser-af-arbejdsmiljoe-sath/.

[32] Kotter J.P. (1996). Leading Change.

